# Intraosseous tibia varix: Unexpected cause of leg pain: A case report

**DOI:** 10.1016/j.ijscr.2025.111834

**Published:** 2025-08-18

**Authors:** Abdullah Almohimeed, Mohamed Achraf Ferjani, Mohamed Ali Bekkay, Khaled Kamoun

**Affiliations:** a HafrAl-Baten Health Cluster, Hafar Al Batin, Saudi Arabia; b University of Tunis El Manar Faculty of Medicine of Tunis, Tunisia

**Keywords:** Perforator vein insufficiency, Chronic leg pain, Intraosseous varices

## Abstract

**Introduction and importance:**

Intraosseous varicose veins are a rare cause of severe lower limb pain, often misdiagnosed due to their atypical presentation. While varicose veins commonly affect superficial vessels, intraosseous involvement remains an unusual finding with limited documentation in medical literature. This report highlights a case of sudden, severe mid-leg pain due to intraosseous venous drainage anomaly.

**Case presentation:**

A 66-year-old male, with no known medical history, presented with an 8-month history of intermittent midshaft left leg pain. The pain began insidiously and gradually increased in frequency and intensity. On examination, there was no bluish discoloration of the medial aspect of the leg with mild swelling. There were no visible dilated veins, scars, or muscle wasting. The patient was able to walk without limping. Imaging studies, including X-ray and MRI were performed. MRI revealed findings consistent with intraosseous venous drainage abnormality.

**Clinical discussion:**

Intraosseous varicose veins are rarely considered in the differential diagnosis of leg pain, contributing to diagnostic delays. MRI is crucial for identifying these vascular anomalies, especially when other investigations are inconclusive. While surgical options like sclerotherapy or vessel ligation exist, conservative management can be effective for select patients.

**Conclusion:**

This case highlights the importance of considering intraosseous varicose veins in patients with unexplained leg pain, even in the absence of typical varicose vein symptoms. MRI is essential for diagnosis, and while conservative treatment may be initially successful, long-term surveillance and consideration of alternative interventions are necessary.

## Highlights

• **Rare Presentation:** Intraosseous tibia varix is an uncommon cause of leg pain, particularly in a 66-year-old male with no prior history of venous insufficiency.
• **Atypical Symptoms:** The patient's intermittent pain, localized to the midshaft of the leg and occurring only after standing, deviated from the typical presentation of varicose veins, making diagnosis challenging.
• **Diagnostic Value of MRI:** MRI played a crucial role in identifying the intraosseous venous anomaly after initial physical examination and radiographs were unremarkable. This highlights the importance of advanced imaging in cases of unexplained limb pain.
• **Conservative Management:** Successful initial management with conservative measures, including analgesics and activity modification, demonstrates a potential non-surgical approach for select patients.
• **Long-Term Surveillance:** The 12-month follow-up, demonstrating reduced pain frequency and the patient's continued preference for conservative treatment, provides valuable insights into the long-term course of this condition.
• **Need for Further Research:** The rarity of intraosseous tibia varix and the variable clinical presentations highlight the need for additional research to better understand its pathophysiology and optimal management strategies.

## 1 Introduction

Unexplained lower limb pain can pose a significant diagnostic challenge, often requiring clinicians to consider rare and unusual etiologies. Intraosseous varicose veins, an infrequently encountered vascular anomaly, represent one such diagnostic conundrum. While varicose veins commonly affect superficial leg veins, their presence within bone is rare and poorly documented [1]. These intraosseous venous abnormalities are rarely described [2], creating diagnostic challenges due to their atypical presentation and lack of clinician awareness. The pathophysiology of pain arising from intraosseous varices remains incompletely understood, with hypotheses including venous hypertension, vessel wall distension, and irritation of the periosteum or medullary canal [3]. This incomplete understanding further complicates the diagnostic process. In some instances, anomalous intraosseous venous drainage may involve abnormally large communicating veins between superficial and intraosseous systems [4], or reflux from the posterior tibial vein tributaries into the bone marrow [2]. This report describes a 66-year-old male with an 8-month history of intermittent midshaft left leg pain. MRI revealed dilated intraosseous veins, prompting consideration of this rare diagnosis. Due to the patient's hesitation to undergo invasive procedures and his reported symptomatic relief after receiving the diagnosis, conservative management was initially chosen. This report highlights the importance of considering intraosseous varicose veins in the differential diagnosis of unexplained lower limb pain and demonstrates the utility of advanced imaging, such as MRI, in their detection [5]. While treatment options like surgical intervention and sclerotherapy exist [6], this case details the outcome of conservative management. The work has been reported in line with the SCARE criteria [7].

## 2 Case report

### 2.1 Patient and observation

#### 2.1.1 Patient information

A 66-year-old male, with no known medical history, presented to the clinic with an eight-month history of intermittent left leg pain. The pain always began after standing up and lasted up to five hours, after which the symptoms completely resolved. The patient reported no allergies to medications or materials. He was a non-smoker and did not consume alcohol. His occupation involved primarily office work, but his symptoms prevented him from standing for extended periods. He reported a moderate level of physical activity otherwise. The patient denied any previous treatments for this leg pain.

#### 2.1.2 Clinical findings

**Fig. 1:**
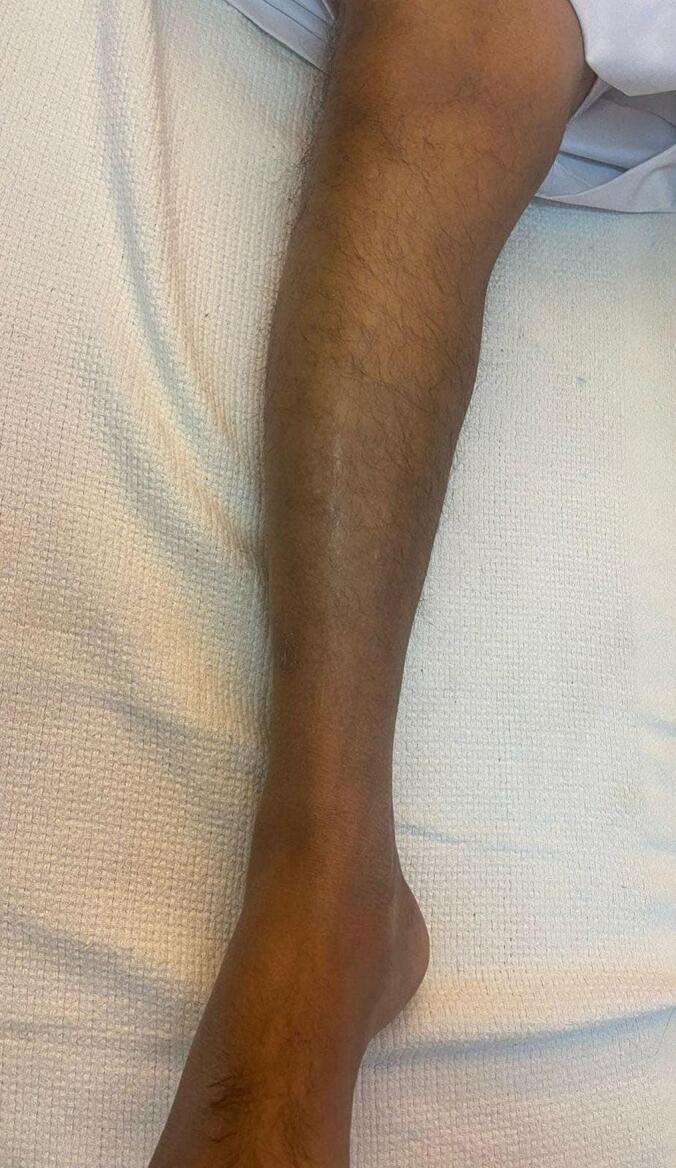
Clinical aspects of the leg that appear without abnormalities.

The pain was located in the midshaft of the left leg and described as intermittent, with a gradual onset, ranging from mild to moderate intensity when initially presenting eight months prior. There was no radiation of the pain. On physical examination, there was no discoloration or varicose pattern over the medial aspect of the leg, but mild swelling was observed. There were no erythema, warmth, or skin changes. The patient denied any numbness, tingling, or paresthesia. Palpation revealed mild tenderness localized over the anteromedial midshaft of the leg, with no palpable mass or crepitus. No visible dilated veins, scars, or muscle wasting were observed (Fig. 1).

The patient's gait was normal, with no limp observed. Neurovascular examination, including assessment of pulses, sensation, and reflexes, was within normal limits.

#### 2.1.3 Timeline of current episode

Eight months prior to presentation, the patient experienced the insidious onset of intermittent, dull, aching pain in the midshaft of his left leg. Initially mild, the pain gradually worsened over several months, increasing in frequency and duration, and eventually preventing ambulation during episodes. The patient began using over-the-counter pain relievers, which provided minimal relief. He presented to the clinic due to the persistent and escalating pain. Imaging studies (X-ray and MRI) were performed, and the patient was diagnosed with intraosseous varicose veins. At 12 months follow-up, the patient continued to experience episodic pain in his left leg, although the frequency and duration of the episodes had decreased compared to the initial presentation. He remained hesitant to pursue surgical intervention, opting to continue with conservative management strategies.

#### 2.1.4 Diagnostic assessment

**Fig. 2:**
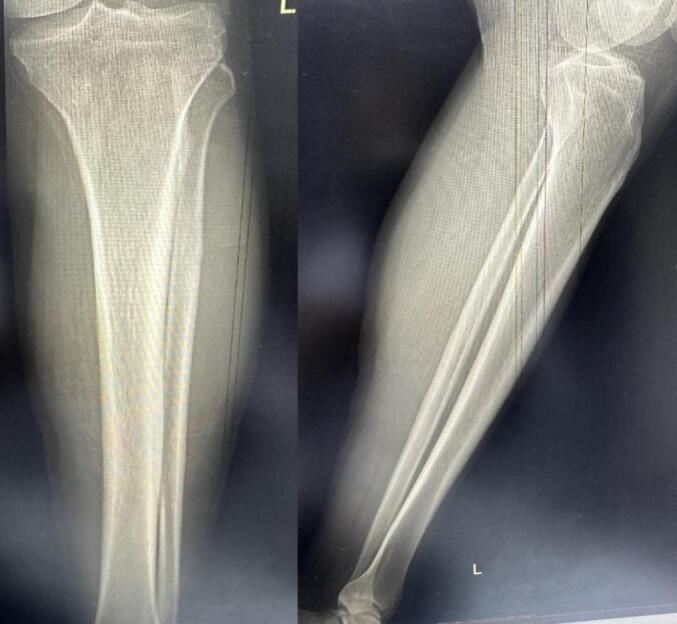
Standard radiographs of the leg revealed no abnormalities.

Initial evaluation included a physical exam, the findings of which are described above. Standard radiographs of the left leg were normal (Fig. 2).

**Fig. 3:**
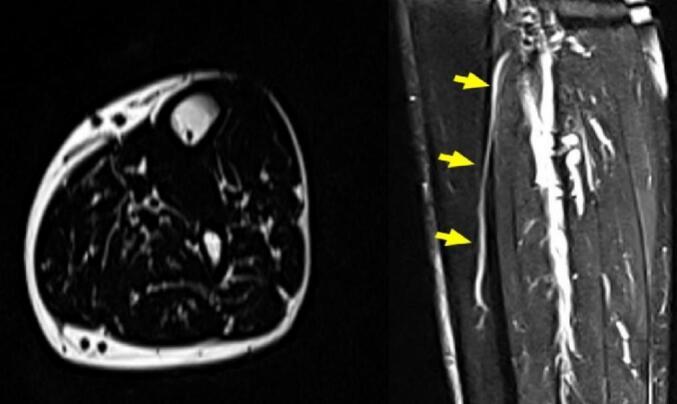
MRI; shows an intraosseous vein within the tibial medullary canal with T2 hyperintensity and posterior cortical communication. No other abnormalities, such as bone marrow edema, soft tissue masses, or stress fractures, were detected.

Laboratory testing, including a complete blood count (CBC), erythrocyte sedimentation rate (ESR), and C-reactive protein (CRP), was within normal limits, ruling out infection or inflammatory processes. Subsequently, magnetic resonance imaging (MRI) of the left leg was performed. The MRI revealed an intraosseous vessel in the pretibial region (Fig. 3). This structure extended from the posterior cortex towards the anterior aspect and was consistent with a prominent intraosseous vein. The lesion demonstrated a hyperintense signal on T2-weighted images and an isointense signal on T1-weighted sequences. There was no enhancement post-contrast, which suggests slow-flowing or stagnant venous blood rather than an aggressive vascular lesion.

#### 2.1.5 Diagnosis

Several diagnoses were considered, including medial tibial stress syndrome and chronic exertional compartment syndrome, but the patient's pain pattern (occurring only after standing), normal physical exam (aside from localized tenderness and mild swelling), and lack of neurological symptoms made these less likely. Normal radiographs ruled out stress fractures. MRI findings of a pretibial intraosseous vessel strongly suggested intraosseous varicose veins, supported by the pain pattern and mild swelling. Conservative management, including analgesics and activity modification, is typically the first line of treatment. However, surgical options like sclerotherapy or vessel ligation may be necessary if conservative measures fail. Prognosis varies depending on the venous abnormality and treatment response.

#### 2.1.6 Therapeutic interventions

Conservative management was initially chosen due to the patient's preference to avoid invasive procedures. This approach focused on symptom relief and minimizing venous congestion. The patient was advised to take analgesics, such as ibuprofen or paracetamol, as needed for pain. Activity modification was a key component of the treatment plan, with the patient instructed to avoid prolonged standing and elevate his legs whenever possible. No compression therapy was initially recommended. After a thorough discussion regarding the nature of his condition, the patient reported partial relief of his symptoms and expressed a better understanding of the rationale for conservative management. He is currently adhering to the prescribed treatment strategy and has elected to continue with conservative measures, including analgesics and activity modification, with ongoing clinical and imaging surveillance. The option of surgical intervention, such as sclerotherapy or surgical ligation, remains a possibility if conservative treatment proves insufficient in the long term.

#### 2.1.7 Follow-up and outcome of interventions

At the 12-month follow-up visit, the patient reported a decrease in the frequency and intensity of his leg pain episodes. Using the Numeric Rating Scale (NRS), he rated his average pain during episodes as 2 out of 10, compared to 4–5 out of 10 at initial presentation. He reported no acute pain episodes in the preceding 3 months. In terms of physical function, the patient was able to stand and walk for up to 45 min without experiencing discomfort, compared to only 10–15 min at baseline. He resumed light physical activity, including short walks and home exercises. He expressed satisfaction with the conservative management approach and elected to continue with activity modification and analgesics as needed, under ongoing clinical and imaging surveillance. He remains hesitant to pursue surgical intervention at this time.

#### 2.1.8 Patient perspective

“Understanding my diagnosis and avoiding prolonged standing has significantly reduced my leg pain. I'm satisfied with the conservative approach so far.”

## 3 Discussion

This case report presents the diagnostic challenges associated with intraosseous varicose veins, a rare vascular anomaly. Schobinger first described this in 1962 in a 66-year-old man with leg swelling and phlebitis due to pretibial intraosseous varices [8]. The pathophysiology remains unclear. One hypothesis, presented by Peh et al. [9], suggests the intraosseous drainage anomaly is pre-existent, with the subcutaneous component developing secondary to increased pressure.

Previous case reports of intraosseous varicose veins, such as those by Rezaie et al. [1] and Peh et al. [9], commonly describe patients presenting with localized leg pain, often associated with visible superficial varices or soft tissue swelling. In contrast, our patient's presentation was atypical: pain was triggered exclusively by standing, without visible varicosities or cutaneous signs, making clinical suspicion difficult.

In terms of management, Peh et al. [9] successfully treated a similar case using imaging-guided sclerotherapy, while Rezaie et al. [1] described significant pain requiring intervention. Our patient, however, opted for a strictly conservative approach.

These comparisons highlight both the variability of presentation and the importance of individualized treatment planning. They also emphasize the diagnostic value of MRI, especially when Doppler ultrasound or X-ray fails to detect abnormalities.

The crucial role of advanced imaging, especially MRI, is emphasized. While Doppler ultrasound excluded superficial and deep venous insufficiency, MRI revealed the pretibial intraosseous vessel, key to the diagnosis [5,7]. This aligns with Ramelet et al.'s [2] description of MRI findings in intraosseous perforating vein incompetence: superficial varicosities, a tibial bone defect, and an enlarged nutrient canal. Our case reinforces the value of MRI in identifying unusual vascular anomalies when other investigations are negative.

Conservative management, including analgesics and activity modification, was chosen based on patient preference, highlighting the importance of individualized treatment [1]. At 12-month follow-up, the patient reported decreased pain frequency and opted to continue conservative measures. While he experienced some relief and understands the rationale for this approach, surgical options like resection [1] or sclerotherapy [2] remain viable if conservative treatment fails.

This case contributes to the limited literature, emphasizing the need for increased clinician awareness of intraosseous varicose veins, particularly given their variable presentation [2]. Further research is crucial to better understand the pathophysiology, diagnosis, and optimal management. As noted by Rezaie et al. [1], the limited number of reported cases contributes to the difficulty in understanding the pathophysiology of pain in this condition. Ramelet et al. [2] also emphasize the need to investigate the origin of intraosseous venous hypertension, especially along the posterior tibial veins. Detailed reports like this case are vital for expanding our knowledge and ultimately improving patient care.

## 4 Conclusion

This case report highlights the diagnostic challenges posed by intraosseous varicose veins, particularly when presenting with atypical features. The absence of superficial varicosities and skin changes, combined with initially unremarkable physical exam and radiographic findings, underscores the importance of a broad differential diagnosis and the need for advanced imaging, like MRI, in cases of persistent unexplained limb pain. While conservative management may provide adequate symptom relief in some cases, as observed in our patient's initial response, the potential for persistent or recurrent symptoms necessitates ongoing surveillance and consideration of alternative interventions, such as sclerotherapy or surgical ligation, if conservative measures prove insufficient. This case contributes to the growing but still limited body of literature on intraosseous varicose veins and emphasizes the need for increased awareness among clinicians and further research to elucidate optimal diagnostic and therapeutic strategies.

## CRediT authorship contribution statement

Patients management: Kamoun Khaled, Mohamed Ali Bekkay and Almohimeed Abdullah. Data collection: Mohamed Achraf Ferjani, Almohimeed Abdullah and Kamoun Khaled. Manuscript drafting: Mohamed Achraf Ferjani. Manuscript revision: Almohimeed Abdullah and Khaled Kamoun. All the authors read and approved the final version of this manuscript.

## Consent to publish

Written informed consent was obtained from the patient for publication and any accompanying images. A copy of the written consent is available for review by the Editor-in-Chief of this journal on request.

## Ethical approval

Ethics approval is not required for case reports in our institution as they are deemed not to be research.

## Guarantor

Mohamed Achraf Ferjani.

## Research registration number

It's a case and a retrospective case.

No section for case report retrospectively study was found in the register provided.

## Funding

The author(s) declared that no grants were involved in supporting this work.

## Declaration of competing interest

No competing interests were disclosed.

## Data Availability

Underlying data. No data are associated with this article.
